# Astaxanthin inhibits cytokines production and inflammatory gene expression by suppressing IκB kinase-dependent nuclear factor κB activation in pre and postpartum Murrah buffaloes during different seasons

**DOI:** 10.14202/vetworld.2018.782-788

**Published:** 2018-06-10

**Authors:** Lakshmi Priyadarshini, Anjali Aggarwal

**Affiliations:** Animal Physiology Division, ICAR-National Dairy Research Institute, Karnal - 132 001, Haryana, India

**Keywords:** astaxanthin, Murrah buffalo, nuclear factor κB p65, summer, winter

## Abstract

**Aim:**

We examined regulatory function of astaxanthin on mRNA expression of nuclear factor κB (NF-κB) p65, interleukin-6 (IL-6), tumor necrosis factor alpha (TNF-α), and interferon gamma (IFN-γ) in peripheral blood mononuclear cells in pre and postpartum Murrah buffaloes during summer (temperature-humidity index [THI]=86; relative humidity [RH]=24) and winter (THI=58.74; RH=73) seasons.

**Materials and Methods:**

A total of 32 Murrah buffaloes apparently healthy and in their one to four parity were selected from National Dairy Research Institute herd and equally distributed randomly into four groups (control and supplemented groups of buffaloes during summer and winter season, respectively). All groups were fed according to the nutrient requirement of buffaloes (ICAR, 2013). The treatment group was supplemented with astaxanthin at 0.25 mg/kg body weight/animal/day during the period 30 days before expected date of calving and up to 30 days postpartum.

**Results:**

There was downregulation of NF-κB p65 gene in all the groups. NF-κB p65 mRNA expression was lower (p<0.05) in treatment than control group from prepartum to postpartum during summer, while mRNA expression was low only on day 21 after calving during winter season. The mRNA expression of IL-6, TNF-α, and IFN-γ was lower (p<0.05) in treatment than a control group of buffaloes during summer and winter seasons. The mRNA expression of NFkB p65, IL-6, TNF-α, and IFN-γ was higher (p<0.05) in summer than in winter seasons.

**Conclusion:**

The xanthophyll carotenoid astaxanthin a reddish-colored C-40 compound is a powerful broad-ranging antioxidant that naturally occurs in a wide variety of living organisms, such as microalgae, fungi, crustaceans, and complex plants. Astaxanthin blocked nuclear translocation of NF-κB p65 subunit and IκBα degradation, which correlated with its inhibitory effect on IκB kinase (IKK) activity. These results suggest that astaxanthin, probably due to its antioxidant activity, inhibits the production of inflammatory mediators by blocking NF-κB activation and as a consequent suppression of IKK activity and IκB-degradation.

## Introduction

Astaxanthin is a lipophilic, pinkish-orange carotenoid (3,3’-dihydroxy-β,β-carotene-4,4’-dione) found in algae, seafood (crustacean shells, crab, shrimps, and fish) by Qin *et al*. [1] and various plants, giving them their exclusive colored aspect [2]. The main source of AST is the microalga *Haematococcus pluvialis*, which contains maximum concentrations [3]. Astaxanthin is also used as a dietary additive in the USA, Japan, South Korea, and Sweden [4]. Like another carotenoid, astaxanthin manifests high protective antioxidant [5,6] and anticancer [7,8] properties which decrease oxidative stress and inflammation [9,10], reduces rethrombosis after thrombolysis [11] and is efficient in ischemia-reperfusion [12], arterial hypertension [13], and dyslipidemia [14].

Nuclear factor κB (NF-κB) is a well-characterized transcription factor that is known to regulate the expression of a wide range of genes, including those of cytokines, chemokines, and cytokine receptors [15] that control various aspects of the immune [16] and inflammatory response [17]. The liberated NF-κB translocates to the nucleus and binds as a transcription factor to kB motifs in the promoters of target genes, leading to their transcription. Aberrant NF-κB activity is associated with various inflammatory diseases, and most anti-inflammatory drugs suppress inflammatory cytokine expression by inhibiting the NF-κB pathway [18,19]. Thus, an NF-κB inhibitor has clinical potential in inflammatory diseases. NF-κB responds to oxidative stress promptly and regulates the transcription of genes involved in a wide range of antioxidant, immune and inflammatory cell functions [20]. NF-κB activation is highly dependent on oxidative stress, which is associated with the immune response [21,22]. An imbalance in oxidants/antioxidants, an excess of oxidants and/or a depletion of antioxidants, can lead to oxidative stress [23]. The inhibition or clearance of intracellular reactive oxygen species generation and accumulation by nicotinamide adenine dinucleotide phosphate oxidase decreased NF-κB activation [24].

We hypothesized that astaxanthin regulated the production of pro-inflammatory cytokines by inhibiting NF-κB activation during different seasons. We studied effect of astaxanthin (at 0.25 mg/kg body weight/animal) as an antioxidant on mRNA expression of NF-κB p65, IL-6, TNF-α, and interferon gamma (IFN-γ) in peripheral blood mononuclear cells (PBMCs) in pre and postpartum Murrah buffaloes during summer and winter seasons.

## Materials and Methods

### Ethical approval

The experiment was approved by the Institutional Animal Ethics Committee (approval no. 41-IAEC-18-10) constituted as per the article 13 of the CPCSEA rules, laid down by Government of India. All the ethical guidelines were followed during the course of the experiment.

### Chemicals

Histopaque 1077 from Sigma Chemical Co. (St. Louis, MO, USA) and Dulbecco’s phosphate buffer saline (DPBS) procured from HiMedia Laboratories Pvt. Ltd., India was used for separation of PBMCs. RNeasy (Cat. No-74104) and RNase-Free DNase Set (Cat No. - 79251) were purchased from QIAGEN Pvt. Ltd., New Delhi, India. Nuclease-free water was purchased from Genetix, Biotech Asia Pvt. Ltd (Fermentas life sciences). Revert Aid First strand cDNA synthesis kit (K1622, Thermo Scientific), Dream Taq Green PCR Master Mix (2X) (K 1082), DyNAmo Color Flash SYBER Green Qpcr kit (F-416XL), and GeneRuler^™^ 100 bp DNA Ladder (50 µg) were purchased from Thermo Fisher Scientific (LSG) Mumbai, India.

### Experimental animals

A total of 32 animals, eight each of pregnant buffalo (*Bubalus bubalis*) having an approximate body weight of 600-650 kg, milk yield of (average) 7.6 kg in their one to four parity 30 days before parturition from Livestock Research Center (LRC), National Dairy Research Institute (NDRI), Karnal, were selected for the experiment. The experiments were carried out during two distinct phases coinciding with two seasons of the year, namely winter (December-February) and summer (April-June). From these 32 animals were further randomly divided equally (8 each) into four groups (control and supplemented groups of buffaloes during summer and winter seasons, respectively). The experimental animals were given adaptation period of 1 week to get adapted to new surroundings. Control groups of buffaloes during summer and winter seasons remained without provision of astaxanthin supplementation, while treatment groups of buffaloes were managed with astaxanthin supplementation during summer and winter seasons, respectively. The astaxanthin as powder form was fed at 0.25 mg/kg body weight/day [25] mixing with concentrate mixture, from 30 days before parturition till 30 days after parturition. At the time of experiment, all the animals were clinically healthy and free from any abnormalities. The experimental animals were maintained and fed as per standard practices followed at LRC, NDRI, and Karnal.

### Blood collection and RNA extraction

About 6-7 ml of fresh blood samples was drawn aseptically from each animal in potassium-EDTA coated Vacutainer tubes (BD-PlymouthPL6 7BP, UK) and was immediately transported to laboratory under refrigeration. Blood samples were collected at 30, 21, 15, and 7 days prepartum, day of calving and 7, 15, and 21 days postpartum from each animal in both seasons.

### Isolation of PBMC

Bovine PBMCs were isolated after blood centrifugation at 3000 rpm at 4°C for 30 min in a refrigerated centrifuge (Sigma, Germany) for separation of lymphocytes. The buffy coat was harvested and re-suspended in 1:1 v/v DPBS. PBMCs were isolated by density gradient centrifugation method using lymphocyte separation medium, Histopaque 1077 (Sigma). The whole content was layered carefully onto the Histopaque to produce a clean interface between the two layers. Further, it was centrifuged at 2000 rpm for 30 min at room temperature. The white opaque mononuclear fraction from the interface was collected between the DPBS and Histopaque. Further, centrifugation was done at 1500 rpm for 7 min for washing the cells with PBS (pH=7.4). Finally, the cell pellet was obtained.

### RNA extraction

Total RNA from PBMC was isolated using RNeasy Mini Kit (Qiagen India Pvt. Ltd.) according to the manufacturer’s protocol. The quality and integrity of isolated RNA was checked by carrying out agarose gel electrophoresis in 1.5% agarose in 1× TAE buffer at 100 Volts for 30 min. The RNA purity was verified by optical density (OD) absorption ratio at λ260/λ280 using Biospec-nano Spectrophotometer (Shimadzu Corp., Japan). A ratio of ~2.0 was generally accepted as “pure” for RNA. The RNA samples with good purity and integrity were used for cDNA synthesis.

### cDNA synthesis

For each sample, about 200 ng of total RNA was used for cDNA synthesis using Revert Aid First strand cDNA synthesis kit (Fermentas, USA) by reverse transcription polymerase chain reaction (PCR) according to the manufacturer’s protocol. The RT reaction was carried out at 25°C for 10 min, 42°C for 60 min, and 75°C for 5 min in a thermal cycler (Bio-Rad, USA).

### Primers

**Figure-1:**
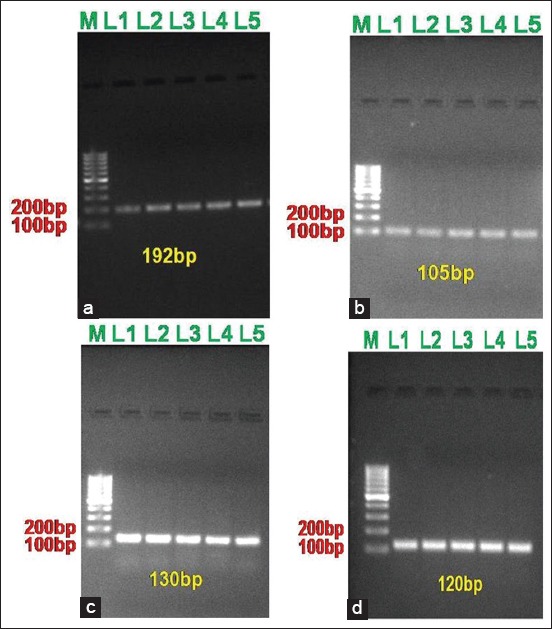
Agarose gel electrophoresis of Real time PCR amplified products of (a) NFkB p65 (192 bp); (b) IL-6 (105 bp); (c) TNF-α (130 bp) and (d) IFN-γ (120 bp) gene products on 1.5% agarose.

Primers for NF-κB, IL-6, TNF-α, and INF-γ were designed using primer 3.0 software or suggested from published literature. The sequences and expected PCR product length are shown in Table-1 and Figure-1.

**Table-1:** Sequence, annealing temperature, and fragment size of the pairs of primers used for RT-PCR.

Gene	Primer sequence	Annealing temp. (°C)	Fragment size (bp)	Accession no.
NF-kB p65 (RELA)	F-TCCAAGTTCCCATAGAAGAGCA R-TCCCAGAGTTCCGATTCACC	57	192	DQ355511
IL-6	F-ATGACGAGTGTGAAAGCAGC R-TCGCCTGATTGAACCCAGAT	59	105	AY347710.1
TNF-a	F-GGTCAACATCCTGTCTGCCA R-ACTGAGGCGATCTCCCTTCT	59	130	XM_006041930.1
Interferon g	F-CGCAAAGCCATAAATGAACTC R-GGACCATTACGTTGATGCTC	60	120	AF484688.1

PCR=Polymerase chain reaction

### Quantitative real-time polymerase chain reaction (RT-PCR)

The RT-PCR reaction was carried out in Applied Biosystems 7500 RT-PCR systems using 0.5 μl of cDNA, 5 μl of Maxima SYBR green qPCR master mix, and 0.5 μl of concerned gene sequence-specific forward and reverse primers (10 pmol), and the final volume of 10 μl was made with nuclease-free water. The RT-PCR program consisted of initial heating at 50°C for 2 min followed by 95°C for 10 min, and samples were amplified for 40 cycles (95°C for 30 s; 58°C for NF-κB, IL-6, TNF-α and INF-γ, 59°C for GAPDH for 30 s, and 72°C for 30 s). The final extension at 72°C incubation was continued for a further 10 min GAPDH was used as housekeeping gene. The relative quantification of target gene was done by the 2^ΔΔCT^ method [26].

### Statistical analysis

The data were analyzed using SAS Software, version 9.3 of the SAS System, Copyright^©^ (2011) SAS Institute Inc., Cary, NC, USA. Data from different experiments are presented as mean±standard error. The pairwise comparison was drawn using Tukey’s multiple comparison tests. The difference at p<0.05 was considered to be statistically significant. The model performed for each test is as follows:

Y_ij_=µ+α_i_+β_j_+H_k_+e_ij_

Where, Y_ij_ is a dependent variable, μ is the overall mean, α_i_ effect of buffaloes (i = 1, 2, 3,…, 16); β_j_ = effect of treatment j (j=astaxanthin supplemented or non-supplemented); H_k_ = effect of time period and e_ij_ is the random error which is assumed to be independent and normally distributed.

## Results

During summer season, the NF-κB p65 gene expression of control and treatment groups was downregulated significantly (p<0.05) on day of calving and on day 21 after calving as compared to day 30 before calving, respectively. The NF-κBp65 gene expression decreased significantly (p<0.05) in treatment group as compared to control group on day 7 before calving, on day of calving and during postpartum period. During winter season, the NF-κBp65 gene expression of control and treatment groups was downregulated significantly (p<0.05) on day of calving and on day 21 after calving as compared to day 30 before calving, respectively. The NF-κB p65 gene expression decreased significantly (p<0.05) in treatment groups as compared to control group on day 60 after calving (Table-2). Gene expression of NF-κB p65 was positively correlated to genes IL-6, TNFα, and INF-γ (Table-3). The NF-κBp65 gene expression was higher (p<0.05) in summer than in winter season.

**Table-2:** Mean (±SE) mRNA expression of NF-kB p65 gene in control and treatment groups of buffaloes during pre and postpartum period in different seasons.

Days relative to calving	Summer		Winter	
	
	Control	Treatment	Control	Treatment
−30	1.00^dA^±0.00	1.00^fA^±0.00	1.00^gA^±0.00	1.00^fA^±0.00
−21	0.23^aA^±0.03	0.21^aA^±0.03	0.19^aA^±0.01	0.13^aA^±0.01
−15	0.61^bA^±0.02	0.67^cdA^±0.03	0.52^bA^±0.02	0.45^bA^±0.03
−7	0.67^bA^±0.03	0.52^bB^±0.02	0.67^cdA^±0.03	0.62^cA^±0.02
0	0.91^cdA^±0.04	0.74^deB^±0.02	0.71^deA^±0.02	0.69^cdA^±0.04
7	0.98^dA^±0.03	0.78^eB^±0.02	0.78^eA^±0.02	0.72^dA^±0.02
15	1.22^eA^±0.03	0.91^fB^±0.04	0.89^fA^±0. 05	0.83^eA^±0.02
21	0.82^cA^±0.02	0.63^cB^±0.02	0.61^bcA^±0.02	0.51^bB^±0.03

a,b,c,d,e,f,g and A,B means with different superscripts differ significantly (p<0.05) in a column and in a row of each season, respectively. NF-kB=Nuclear factor kB, SE=Standard error

**Table-3:** Correlation among various genes.

	NF-kB p65	IL-6	TNF-α	Interferon γ
NF-kB p65	1			
IL-6	0.304**	1		
TNF-a	0.346**	0.687**	1	
Interferon g	0.695**	0.536**	0.630**	1

** p<0.01. NF-kB=Nuclear factor kB, IL-6=Interleukin-6, TNF-a=Tumor necrosis factor alpha

During the summer season, the IL-6 gene expression in control and treatment groups increased significantly (p<0.05) on the day of calving and day 21 after calving as compared to day 30 before calving, respectively. The IL-6 gene expression decreased significantly (p<0.05) in treatment groups as compared to control group of respective days. During the winter season, the IL-6 gene expression in control and treatment groups increased significantly (p<0.05) on the day of calving and day 21 after calving as compared to day 30 before calving, respectively. The IL-6 gene expression decreased significantly (p<0.05) in treatment groups as compared to control group on day 15, 7 before calving and on day 15, 21 after calving (Table-4). The IL-6 gene expression was higher (p<0.05) in summer than in winter season. IL-6 gene expression was positively correlated to genes NF-κB p65, TNFα, and IFN*γ (Table-3).

**Table-4:** Mean (±SE) mRNA expression of IL-6 gene in control and treatment groups of buffaloes during pre and postpartum period in different seasons.

Days relative to calving	Summer		Winter	
	
	Control	Treatment	Control	Treatment
−30	1.00^aA^±0.00	1.00^aA^±0.00	1.00^aA^±0.00	1.00^aA^±0.00
−21	1.08^aA^±0.03	1.03^aA^±0.02	1.04^aA^±0.02	1.01^aA^±0.01
−15	1.43^bA^±0.03	1.32^bA^±0.03	1.28^bA^±0.02	1.15^bB^±0.02
−7	2.71^eA^±0.02	2.14^eB^±0.02	2.06^dA^±0.03	1.93^dB^±0.03
0	4.13^fA^±0.02	3.64^gB^±0.03	3.32^fA^±0.02	3.21^fA^±0.03
7	2.68^eA^±0.02	2.48^fB^±0.03	2.21^eA^±0.02	2.10^eA^±0.02
15	2.16^dA^±0.03	2.02^dB^±0.03	2.03^dA^±0. 03	1.89^dB^±0.03
21	1.82^cA^±0.03	1.69^cA^±0.03	1.79^cA^±0.03	1.65^cB^±0.03

a,b,c,d,e,f,g and A,B means with different superscripts differ significantly (p<0.05) in a column and in a row of each season, respectively. IL-6=Interleukin-6, SE=Standard error

During the summer season, the TNF-α gene expression in control and treatment groups increased significantly (p<0.05) on the day of calving and day 21 after calving as compared to day 30 before calving, respectively. The TNF-α gene expression was decreased significantly (p<0.05) in the treatment group as compared to control group. During the winter season, the TNF-α gene expression in control and treatment groups increased significantly (p<0.05) on the day of calving and day 21 after calving as compared to day 30 before calving, respectively. The TNF-α gene expression decreased significantly (p<0.05) in the treatment group as compared to control group on day 15^th^ before calving to day 15^th^ after calving, respectively (Table-5). The TNF-α expression was higher (P<0.05) in summer than in winter season. TNF-α gene expression was positively correlated to genes NF-κB p65, IL-6, IFN-γ.

**Table-5:** Mean (±SE) mRNA expression of TNF-a gene in control and treatment groups of buffaloes during pre and postpartum period in different seasons.

Days relative to calving	Summer		Winter	
	
	Control	Treatment	Control	Treatment
−30	1.00^aA^±0.00	1.00^aA^±0.00	1.00^aA^±0.00	1.00^aA^±0.00
−21	1.13^bA^±0.02	1.10^aA^±0.03	1.08^aA^±0.03	1.03^aA^±0.02
−15	2.43^dA^±0.03	1.83^bB^±0.03	1.70^bA^±0.03	1.51^bB^±0.03
−7	2.78^eA^±0.02	1.97^cB^±0.03	1.95^dA^±0.03	1.67^cB^±0.03
0	3.12^fA^±0.02	2.85^dB^±0.02	2.10^eA^±0.03	1.93^dB^±0.03
7	4.91^gA^±0.02	3.71^eB^±0.03	3.14^fA^±0.03	2.92^eB^±0.02
15	2.37^dA^±0.03	2.03^cB^±0.03	2.15^eA^±0.03	1.91^dB^±0.03
21	2.10^cA^±0.03	1.74^bB^±0.03	1.54^bA^±0.03	1.41^bA^±0.03

a,b,c,d,e and A,B means with different superscripts differ significantly (p<0.05) in a column and in a row of each season, respectively. TNF-a=Tumor necrosis factor alpha, SE=Standard error

During the summer season, the IFN-γ gene expression in control and treatment groups was upregulated significantly (p<0.05) from day of calving to day 15 then downregulated significantly (p<0.05) on day 21 after calving as compared to day 30 before calving, respectively. The IFN-γ gene expression decreased significantly (p<0.05) in treatment groups as compared to control group from day 7 before calving to day 21 after calving. During the winter season, the IFN-γ gene expression of control and treatment groups was significantly (p<0.05) upregulated on day of calving to day 15 then downregulated significantly (p<0.05) on day 21 after calving as compared to day 30 before calving, respectively (Table-6). The IFN-γ gene expression decreased significantly (p<0.05) in treatment groups as compared to control group from the day of calving to day 15 after calving. The IFN-γ gene expression was higher (p<0.05) in summer than winter season. IFN-γ gene expression was positively correlated to genes NF-κB p65, TNFα, and IL-6 (Table-3).

**Table-6:** Mean (±SE) mRNA expression of interferon g gene in control and treatment groups of buffaloes during pre and postpartum period in different seasons.

Days relative to calving	Summer		Winter	
	
	Control	Treatment	Control	Treatment
−30	1.00^dA^±0.00	1.00^eA^±0.00	1.00^eA^±0.00	1.00^eA^±0.00
−21	0.07^aA^±0.02	0.04^aA^±0.01	0.09^aA^±0.02	0.02^aA^±0.00
−15	0.31^bA^±0.05	0.28^bA^±0.04	0.26^bA^±0.04	0.14^bA^±0.04
−7	0.78^cA^±0.07	0.52^cB^±0.02	0.51^cA^±0.03	0.43^cA^±0.03
0	1.91^eA^±0.03	1.64^fB^±0.03	1.71^fA^±0.03	1.33^fB^±0.03
7	2.71^fA^±0.02	2.03^gB^±0.03	2.62^gA^±0.06	2.06^gB^±0.04
15	2.92^gA^±0.02	2.15^hB^±0.02	2.83^hA^±0.02	2.62^hB^±0.06
21	0.91^cdA^±0.04	0.71^dB^±0.02	0.73^dA^±0.07	0.65^dA^±0.02

a,b,c,d,e,f,g,h and A,B means with different superscripts differ significantly (p<0.05) in a column and in a row of each season, respectively. SE=Standard error

## Discussion

NF-κB p65 is one of the most important pathways in inflammation and tumors [27]. In our experiment, the mRNA expression of NF-κB p65, IL-6, TNFα, and IFN-γ significantly (p<0.05) decreased in astaxanthin supplemented groups than that of control groups in summer and winter seasons. Astaxanthin decreased the expression of pro-inflammatory mediators such as TNF-α by suppressing IκB-dependent NF-κB activation in both primary macrophages and lipopolysaccharide (LPS)-stimulated RAW264.7 cells [28]. Astaxanthin remarkably suppressed the expression of the mRNA expression of inflammatory mediators, i.e., tumor necrosis factor alpha (TNF-α), IL-6, and in THP-1 macrophages [29]. Astaxanthin significantly decreased the production of IL-6 and TNF-α in LPS-stimulated neutrophils [30]. Astaxanthin treatment reduced the secretion of IL-6 and TNF-α in H_2_O_2_-stimulated U937 mononuclear cells, and this property was elicited by a restoration of the basal SHP-1 protein expression level and reduced NF-κB (p65) nuclear expression [31]. Another study reported that on macrophage NF-κB activation inhibition might be due to antioxidant property of astaxanthin [32]. IL-6 and TNF-α gene expression decreased (p<0.05) after furrowing and in lactation [33]. IFN-γ expression upregulated significantly (p<0.05) from day of calving to day 15 then after was downregulated significantly (p<0.05) on day 21 after calving as compared to day 30 before calving. A study on rat found that astaxanthin inhibits inducible nitric oxide synthase enzyme activity, which decreases the production of nitric oxide as well as prostaglandin E2 and TNF-α [34].

IFN-γ expression increased gradually from prepartum (0.56-fold) up to 2^nd^ week (2.89-fold) postpartum and subsequently decreased in the 3^rd^ (0.73-fold) and 4^th^ week (0.70-fold) in healthy buffaloes [35]. IL-6 and IFN-γ mRNA expressions in liver were higher in astaxanthin fed male broiler chickens (*Gallus gallus*) compared to that of control birds [36].

## Conclusion

We showed that NF-κB inhibited *in* *vivo* production of pro-inflammatory cytokines, such as TNF-α, IL-6, and IFN-γ. Furthermore, it suppressed NF-κB activation may be by inhibiting IKK activity. These results support the idea that astaxanthin prevents inflammatory processes by blocking the expression of pro-inflammatory genes as a consequence of suppressing NFkB activation.

## Authors’ Contributions

LP carried out the research under the supervision of AA. LP collected blood samples, did molecular work and analyzed the data. The manuscript was drafted and revised by LP under the guidance of AA. Both authors read and approved the final manuscript.
